# Overexpression of the HCN2 channel increases the arrhythmogenicity induced by hypokalemia

**DOI:** 10.1007/s12576-019-00684-7

**Published:** 2019-05-13

**Authors:** Kensuke Oshita, Yuko Kozasa, Yasuaki Nakagawa, Yoshihiro Kuwabara, Koichiro Kuwahara, Taku Nakagawa, Noriyuki Nakashima, Teruyuki Hiraki, Makoto Takano

**Affiliations:** 1 Department of Physiology, Kurume University School of Medicine, 67 Asahi-Machi, Kurume, 830-0011 Japan; 2 Department of Anesthesiology, Kurume University School of Medicine, Kurume, Japan; 3 Department of Cardiovascular Medicine, Kyoto University Graduate School of Medicine, Kyoto, Japan; 4 Department of Cardiovascular Medicine, Shinshu University School of Medicine, Matsumoto, Japan

**Keywords:** Hypokalemia, Arrhythmia, HCN2, Heart failure

## Abstract

Hypokalemia, an abnormally low level of potassium (K^+^), is a electrolyte imbalance that commonly occurs in heart failure patients. Hypokalemia is well known to induce lethal ventricular arrhythmia. However, the effects of hypokalemia in failing hearts that have undergone electrophysiological remodeling, i.e., the reactivation of fetal-type ion channels, remain unexplored. We have examined the effect of hypokalemia in the myocytes of transgenic mice overexpressing the hyperpolarization-activated, cyclic nucleotide-sensitive (HCN) channel in the heart (HCN2-Tg mice). Perfusion with a mild hypokalemic solution containing 3 mM K^+^ induced ectopic ventricular automaticity in 55.0% of HCN2-Tg mouse myocytes. In the remaining HCN2-Tg mouse myocytes, the resting membrane potential (RMP) was more depolarized than that of wild-type myocytes subjected to the same treatment and could also be hyperpolarized by an HCN channel blocker. We conclude that in hypokalemia in our mice model, the HCN2 channel was constitutively activated at the hyperpolarized RMP, thereby destabilizing the electrophysiological activity of ventricular myocytes.

## Introduction

Hypokalemia, a type of pro-arrhythmic electrolyte disturbance characterized by an abnormally low level of potassium (K^+^), is frequently observed in patients with cardiovascular disease [1, 2]. Heart failure patients in particular often receive diuretics, some of which can cause an increase in the elimination of K^+^ in the urine, leading to hypokalemia. Almost 50% of heart failure patients die suddenly, most probably due to ventricular arrhythmia. Therefore, it is important to control the serum potassium homeostasis during the treatment of heart failure patients with arrhythmia [3, 4].

The mechanism of ventricular arrhythmia in heart failure is not fully understood. Calcium (Ca^2+^) overload and pathological Ca^2+^ release from the sarcoplasmic reticulum is thought to induce inward Na^+^–Ca^2+^ exchanger currents, giving rise to delayed after-depolarization [5]. Electrophysiological remodeling, i.e., reactivation of fetal cardiac genes, including T-type Ca^2+^ channel and hyperpolarization-activated, cyclic nucleotide-sensitive (HCN) cation channels (HCN2 and -4) have also been reported to increase the vulnerability to arrhythmia [6, 7].

Hypokalemia hyperpolarizes the resting membrane potential (RMP) and prolongs the action potential duration (APD) of the ventricular myocytes [8]. Therefore, it appears likely that HCN2 channels, when re-expressed in the hypertrophied heart, may be activated at a hyperpolarized RMP under hypokalemic conditions and may thereby increase the arrhythmogenicity. We have previously reported that β-adrenergic stimulation induced ectopic ventricular automaticity in the hearts of transgenic mice overexpressing HCN2 (HCN2-Tg mice) [9]. In the present study, our aim was to examine the effects of hypokalemia on the ventricular myocytes isolated from HCN2-Tg mice. We also raised the HCN2-Tg mice on a K^+^-free diet, and compared their electrocardiograms (ECGs) with those of their wild-type (WT) littermates. We will demonstrate that HCN2 overexpression increases the vulnerability to arrhythmia under hypokalemic conditions. Preliminary results of the study reported here have been communicated to the annual meeting of the Physiological Society of Japan [10].

## Methods

### Experimental animals

To generate the HCN2-Tg mice, the cDNA of murine HCN2 was overexpressed in C57BL/6 mice using the alpha-MHC promoter (thereby generating HCN2-Tg mice), as reported previously [7]. We used the HCN2-Tg mice and their WT littermates aged between 10 and 30 weeks of age. The mice were raised in air-conditioned rooms at 25 °C, under a 12/12-h light/dark cycle.

### Cell isolation

After the mice were deeply anesthetized with 5% sevoflurane, their hearts were quickly removed and perfused using a Langendorff apparatus. Single ventricular myocytes were obtained following collagenase digestion, as previously reported [9].

### Electrophysiological measurements

The membrane current and action potentials (APs) were recorded using the ruptured whole-cell patch method with the Axopatch 200B amplifier and Digidata 1320 interface (Molecular Devices, San Jose, CA, USA). The electrode resistance of the patch pipette was 3–4 MΩ when filled with an internal, high-K^+^ solution containing 110 mM aspartic acid, 30 mM KCl, 5 mM K_2_ATP, 5 mM Na_2_ creatine phosphate, 0.1 mM Na_2_GTP, 5 mM EGTA, 1 mM MgCl_2_, and 5 mM HEPES (the pH was adjusted to 7.2 with KOH). The final K^+^ concentration of the internal pipette solution was 159.6 mM. The whole-cell patch was established in the bathing solution which contained 140 mM NaCl, 5.4 mM KCl, 0.5 mM MgCl_2_, 1.8 mM CaCl_2_, and 5 mM HEPES (the pH was adjusted to 7.4 with NaOH) at 33–34 °C. Ivabradine (IVA; Santa Cruz Biotechnology, Inc., Dallas, TX, USA) stock solution (10 mg/ml) was prepared in distilled H_2_O and diluted to a concentration of 10 μM in the bathing solution.

### ECG recording

During the ECG recording, the mice were anesthetized using 3.0% sevoflurane, and their body temperature was monitored and maintained at 37 °C using a warming device. We recorded two-lead ECGs (lead I and lead II) on the WT and HCN2-Tg mice that had been raised on a K^+^-free diet (Research Diets Inc., New Brunswick, NJ, USA) for 6 weeks. IVA (7 mg/kg per day) was dissolved in the drinking water that was supplied to the mice ad libitum for 6 weeks. The ECG waveforms were analyzed using the PowerLab Data Acquisition System and the LabChart software (ADInstruments Inc., Dunedin, New Zealand), as reported previously [9]. At the end of the ECG recordings, the mice were deeply anesthetized with 5.0% sevoflurane and then euthanized by aspirating the blood from the left ventricle. The blood was kept on ice for 15 min, and the serum was obtained by centrifuging the blood samples at 1000 *g* for 5 min at 4 °C. The serum K^+^ concentration was measured using the ion-selective electrode method (Oriental Bio Inc., Gyeonggi-do, South Korea).

### Statistical analysis

The results are expressed as the mean ± standard deviation (SD). Statistical analysis was carried out using Students’ paired *t* test, unpaired *t* test, and the χ^2^ test.

## Results

### Spontaneous action potentials of HCN2-Tg mouse myocytes following induction with mild hypokalemic solution

**Fig. 1:**
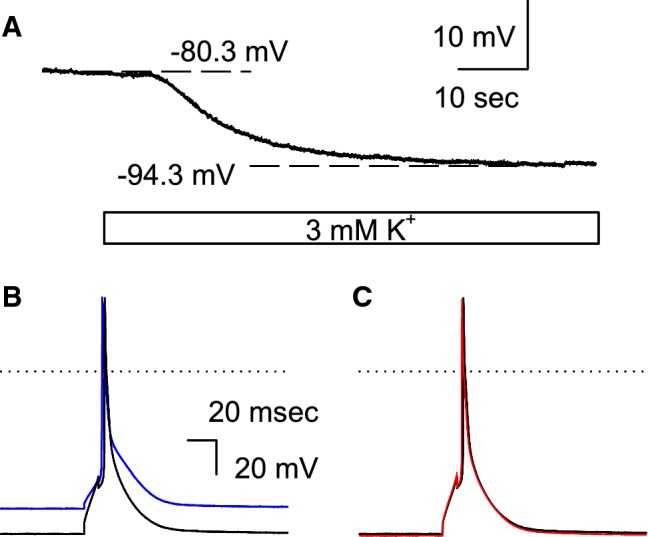
Effect of mild hypokalemia on wild-type (WT) mouse myocytes. **a** Hyperpolarization of the resting membrane potential (RMP) of WT mouse myocytes. As indicated by the blue bar, the bathing solution was switched from the 5.4 mM extracellular potassium (K^+^) concentration ([K^+^]_o_) solution to the 3 mM [K^+^]_o_ solution. **b** The induced action potention (AP) in the 5.4 mM [K^+^]_o_ solution (blue line) and the 3 mM [K^+^]_o_ solution (black line). **c** The AP recorded in the same myocytes as those mentioned in **b** (the black line, control; the red line, in the presence of 10 μM ivabradine [IVA]). The K^+^ concentration of the bathing solution was 3 mM. The dotted line indicates 0 mV (color figure online)

In ventricular myocytes, the RMP is primarily determined by the inward rectifier K^+^ current (*I*_K1_) flowing through the Kir2.1 channel. As shown in Fig. 1a, the RMP of ventricular myocytes isolated from WT mice was hyperpolarized following the equilibrium potential for K^+^ (*E*_K_) when the bathing solution was switched from a normal Tyrode solution (extracellular K^+^ concentration [K^+^]_o_ = 5.4 mM) to a clinically relevant, mild hypokalemic solution ([K^+^]_o_ = 3 mM). No spontaneous action potential (SAP) was observed under this condition (0 of 11 cells). We then induced the AP by current injection and examined the effects of the 3 mM [K^+^]_o_ solution. Unlike the case for myocytes from guinea pigs or rabbits, the APD of murine ventricular myocytes at 90% repolarization (APD_90_) was not markedly prolonged in the hypokalemic solution, when the AP was recorded using the ruptured whole-cell patch method with a pipette solution containing EGTA, a specific chelator for Ca^2+^, as shown in Fig. 1b (blue line, in 5.4 mM [K^+^]_o_; black line, in 3 mM [K^+^]_o_). This was presumably due to the voltage-gated K^+^ channels comprising the “repolarization reserve” of murine myocytes being less sensitive to hypokalemia (for detailed information, see section Limitations of the present study in the Discussion section). We further examined the effects of 10 μM IVA (HCN channel blocker) on the AP. It is evident from Fig. 1c that the waveforms of APs in the presence (red line) and absence (black line) of IVA are superimposable. As summarized in Table 1, the application of IVA showed no significant effects on the RMP or APD_90_ of the WT myocytes.

**Table 1:** The summary of the parameters of the induced action potential in wild-type myocytes

WT	5.4 mM [K^+^]_o_	3 mM [K^+^]_o_	3 mM [K^+^]_o_ + IVA
RMP (mV)	− 81.5 ± 1.0	− 95.0 ± 2.2	− 94.5 ± 1.1
APD_90_ (ms)	30.4 ± 1.8	31.5 ± 1.2	30.9 ± 1.1

The summaries of RMP and APD_90_ in the 5.4 mM [K^+^]_o_, 3 mM [K^+^]_o_, and 3 mM [K^+^]_o_ solutions, all of which contain 10 μM IVA (*n  *= 6)

*APD*_90_ Action potential duration at 90% repolarization,* IVA* ivabradine, [*K*^+^]_o_ extracellular K^+^ concentration, *RMP* resting membrane potential

**Fig. 2:**
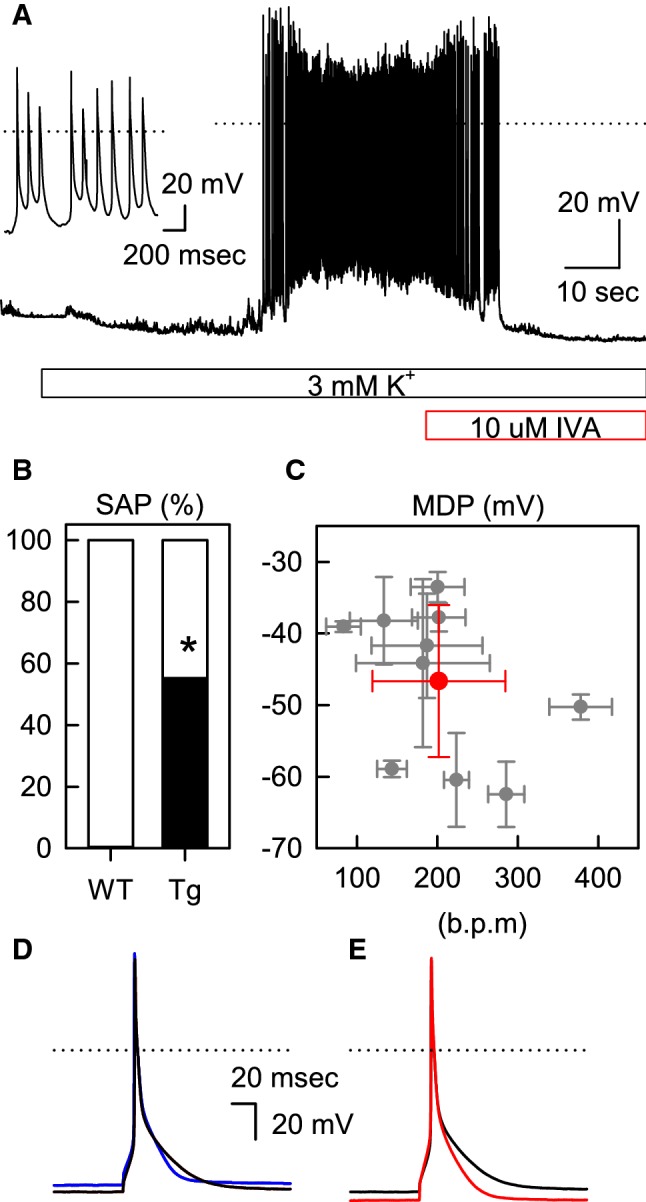
Hypokalemia-induced spontaneous action potentials (*SAPs*) in the myocytes of transgenic mice overexpressing the hyperpolarization-activated, cyclic nucleotide-sensitive (HCN) channel in the heart (HCN2-Tg mice). **a** The perfusion with the 3 mM [K^+^]_o_ solution is shown by the blue bar. The RMP in the 5.4 mM [K^+^]_o_ solution was − 81.4 mV. In the 3 mM [K^+^]_o_ solution, the RMP was hyperpolarized to − 86.0 mV, followed by the generation of SAPs. The red bar indicates the perfusion with 10 μM IVA. After the termination of the SAPs, the RMP was − 90.4 mV. The inset shows the expanded trace of the SAPs. **b** The incidences of SAPs (black portion of bar) in the 3 mM K^+^ solution: WT, 0% (0 of 11 cells); HCN2-Tg, 55.0% (11 of 20 cells). Asterisk indicates that difference is statistically significant at *p  *< 0.01 (χ^2^ test). **c** The summary of firing rates (FRs) and maximal diastolic potentials (*MDPs*) of the SAPs. The gray symbols were obtained in each myocyte by averaging 10 successive SAPs. The red symbol indicates the mean values of the MDP (− 46.6 ± 10.6 mV) and the FR (201.8 ± 82.6 bpm) averaged from 10 myocytes. **d** The induced AP of HCN2-Tg mouse myocytes in the 5.4 mM [K^+^]_o_ solution (blue line) and 3 mM [K^+^]_o_ solution (black line). The RMP and action potential duration at 90% repolarization (APD_90_) were − 83.8 mV and 31.0 ms, respectively, in the 5.4 mM [K^+^]_o_ solution and − 88.1 mV and 36.8 ms, respectively, in the 3 mM [K^+^]_o_ solution. **e**. The effect of 10 μM IVA on the AP in the 3 mM [K^+^]_o_ solution. The AP was recorded in the same HCN2-Tg mouse myocytes as those shown in **d** (the black line, control; the red line, with 10 μM IVA). The application of IVA hyperpolarized the RMP from − 88.1 to − 94.0 mV and shortened the APD_90_ from 36.8 to 29.8 ms (color figure online)

We previously reported that the RMPs of HCN2-Tg mouse myocytes were not significantly different from those of WT mouse myocytes in normal Tyrode solution, but that they were depolarized by β-adrenergic stimulation, followed by the generation of SAPs [9]. Likewise, in 55.0% of the HCN2-Tg mouse myocytes examined, SAPs were immediately induced after perfusion with the 3 mM [K^+^]_o_ bathing solution (11 of 20 cells; Fig. 2a, b); the application of 10 μM IVA successfully terminated the SAPs induced by the 3 mM [K^+^]_o_ solution. The inset of Fig. 2a shows the expanded tracing of the SAPs. In each myocyte, the maximal diastolic potentials (MDPs) and the firing rates (FRs) were calculated from ten successive SAPs; these are summarized in Fig. 2c (gray symbols). The averaged value of MDPs and FRs are indicated by the red symbol in Fig. 2c.

When HCN2-Tg mouse myocytes showed no SAPs in the 3 mM [K^+^]_o_ solution, we induced APs by current injection. Unlike the WT mouse myocytes, the magnitude of hyperpolarization induced by the hypokalemic solution was smaller, as shown by the blue line (in 5.4 mM solution) and the black line (in the 3 mM [K^+^]_o_ solution) in Fig. 2d. Furthermore, the application of 10 μM IVA consistently hyperpolarized the RMP of HCN2-Tg mouse myocytes and shortened the APD, as shown in Fig. 2e (the black line, control; the red line, 10 μM IVA). The effect of the hypokalemic solution and 10 μM IVA on the RMPs and APD_90_ of HCN2-Tg mouse myocytes is summarized in Table 2. Under the hypokalemic condition, the RMP was significantly hyperpolarized by IVA (*p  *< 0.05; *n  *= 6); at the same time, IVA significantly shortened the APD_90_ (*p * < 0.05; *n* = 6). These findings strongly suggest that HCN2 channels overexpressed in the ventricular myocytes were constitutively activated at the RMP and participated as components of the repolarization reserve of HCN2-Tg mouse myocytes under hypokalemic conditions.

**Table 2:** Summary of the parameters of induced action potentials in the myocytes of transgenic mice overexpressing the HCN2 channel in the heart

HCN2-Tg	5.4 mM [K^+^]_o_	3 mM [K^+^]_o_	3 mM [K^+^]_o_ + IVA
RMP (mV)	− 82.1 ± 2.4	− 91.2 ± 3.3	− 93.4 ± 2.9*
APD_90_ (ms)	31.5 ± 1.6	37.1 ± 3.1	30.1 ± 2.2*

The summaries of RMP and APD_90_ in the 5.4 mM [K^+^]_o_, 3 mM [K^+^]_o_, and 3 mM [K^+^]_o_ solutions, respectively, all of which contain 10 μM IVA

*HCN2-Tg* Transgenic mice overexpressing the hyperpolarization-activated, cyclic nucleotide-sensitive (HCN) channel in the heart

*Significant difference at *p * < 0.05 (paired *t* test) in the presence and absence of IVA (*n* = 6)

### The background membrane currents of HCN2-Tg mouse myocytes in a hypokalemic solution

**Fig. 3:**
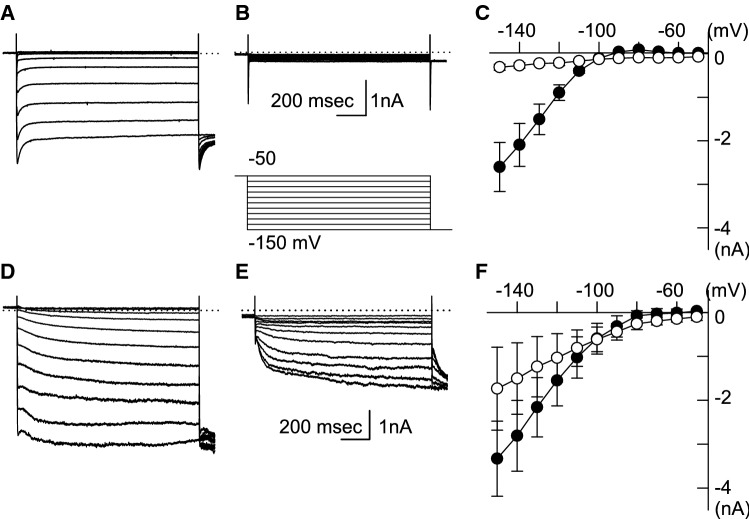
The current–voltage (*I*–*V*) relationships of WT and HCN2-Tg mouse myocytes in 3 mM K^+^ solution. **a** The current traces of WT myocytes recorded in the control condition. **b** The current traces of WT myocytes in the presence of 1 mM barium (Ba^2+^). The inset shows the pulse protocol. **c** The *I*–*V* relationships of the background current of WT mouse myocytes. The amplitudes of the background current were measured at the end of the hyperpolarizing pulses: filled circle, control; open circle, in the presence of 1 mM Ba^2+^. **d** The current traces of HCN2-Tg myocytes in the cotrol condition. **e** The current traces of HCN2-Tg myocytes in the presence of 1 mM Ba^2+^. **f** The *I-V* relationships measured in HCN2-Tg myocytes (same conditions as in **c**)

We had previously reported that neither the amplitudes of *I*_K1_ nor the Kir2.1 mRNA levels of the WT and HCN2-Tg mouse myocytes were different [9]. Nevertheless, the RMP of HCN2-Tg mouse myocytes was more depolarized under hypokalemic conditions. In order to explore the ionic mechanisms of RMP depolarization under hypokalemic conditions, we compared the background currents of the WT and HCN2-Tg mouse myocytes in the 3 mM [K^+^]_o_ solution. As shown in Fig. 3, hyperpolarizing pulse steps ranging from − 50 to − 150 mV were applied, and the amplitudes of the membrane currents were measured at the end of the test pulses. In WT mouse myocytes, small outward components of the *I*_K1_ were observed at the membrane potentials that were more positive than − 90 mV (Fig. 3a). The current–voltage (*I*–*V*) relationship intersected with the abscissa at approximately − 95 mV (filled circle in Fig. 3c). When the *I*_K1_ was inhibited by 1 mM barium (Ba^2+^), no time-dependent currents were observed in WT mouse myocytes (Fig. 3b), indicating a linear *I*–*V* relationship (open circle in Fig. 3c).

In HCN2-Tg mouse myocytes, the hyperpolarizing pulses activated both the *I*_HCN_ and *I*_K1_, as shown in Fig. 3d. In the *I*–*V* relationships measured in the 3 mM [K^+^]_o_ solution, the outward components of the *I*_K1_ were almost cancelled out by the overlapping activation of the HCN-driven current (*I*_HCN_) between − 80 and − 60 mV (filled circle in Fig. 3f). The application of 1 mM Ba^2+^ disclosed a robust activation of *I*_HCN_, as shown in Fig. 3e. The *I*–*V* relationship measured in the presence of Ba^2+^ showed a clear inward-rectification at the membrane potentials more negative than − 70 mV, which was due to the activation of the *I*_HCN_ (open circle in Fig. 3f). These *I*–*V* relationships comprehensively explained the unstable, depolarized RMP of HCN2-Tg mouse myocytes in 3 mM [K^+^]_o_ solution.

### The activation curves of *I*_HCN_ in hypokalemic solution

It is well known that extracellular K^+^ has multiple effects on the biophysical properties of *I*_HCN_ [11]. First, K^+^ itself is highly permeable through HCN channels. The permeability ratio for K^+^ and Na^+^ (*P*_Na_/*P*_K_) was 0.20–0.25, depending on the extracellular K^+^ concentration. Second, extracellular K^+^ is an activator of *I*_HCN_, maintaining the ionic conductance through the HCN channels. Third, extracellular K^+^ modulates the voltage-dependence of *I*_HCN_ activation when the K^+^ concentration of the bathing solution is increased to non-physiological levels (i.e., 30 or 100 mM).

**Fig. 4:**
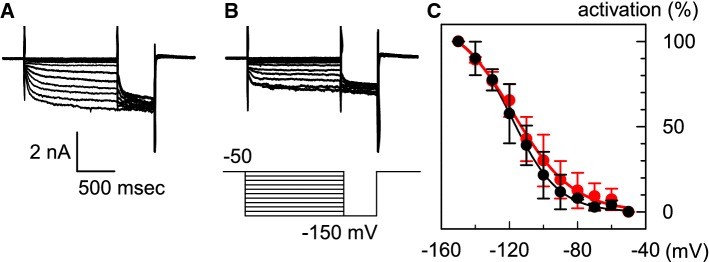
The activation curves of *I*_HCN_ in 5.4 mM [K^+^]_o_ and 3 mM [K^+^]_o_ bathing solution. **a***I*_HCN_ recorded in the 5.4 mM [K^+^]_o_ solution; *I*_K1_ was suppressed by 1 mM Ba^2+^. **b***I*_HCN_ in the 3 mM [K^+^]_o_ solution. The *I*_HCN_ was recorded in the same HCN2-Tg mouse myocytes as those mentioned in **a**. The pulse protocol is indicated below the traces. **c** The activation curves of *I*_HCN_ in the 5.4 mM [K^+^]_o_ solution (filled black circles) and in 3 mM [K^+^]_o_ solution (filled red circles). The *V*_1/2_ (membrane potential for half-maximal activation) was − 118.5 ± 2.9 mV (in the 5.4 mM [K^+^]_o_ solution) and − 117.7 ± 3.4 mV (in the 3 mM [K^+^]_o_ solution) (*n  *= 6) (color figure online)

Therefore, we examined the voltage dependence of *I*_HCN_ in HCN2-overexpressing ventricular myocytes under hypokalemic conditions. *I*_HCN_ was recorded in the same myocytes immersed in a 5.4 mM [K^+^]_o_ solution (Fig. 4a) and in a 3 mM [K^+^]_o_ bathing solution (Fig. 4b). In these experiments, *I*_K1_ was suppressed by the addition of 1 mM Ba^2+^. The amplitude of *I*_HCN_ decreased under hypokalemic conditions; at − 150 mV, the amplitude of *I*_HCN_ in the 3 mM [K^+^]_o_ bathing solution was 55.6 ± 13.9% of that in the 5.4 mM [K^+^]_o_ normal Tyrode solution (*n  *= 6). In order to obtain the voltage-dependent activation curve, we applied conditioning pulses (from − 50 to − 150 mV), followed by the test pulse (− 150 mV). The amplitudes of the time-dependent components during the test pulse were normalized by the maximal value; these are plotted in Fig. 4c. The black and red symbols in Fig. 4c indicate the activation curves obtained in the 5.4 mM [K^+^]_o_ solution and 3 mM [K^+^]_o_ solution, respectively. The lines in the case of each group of data were fitted using the Boltzmann’s equation:

$$\%{\text{activation}}=1/(1+\exp(({V_{\text{m}}}-V_{1/2})/s)),$$

where *V*_m_ is the membrane potential; *V*_1/2_ is the membrane potential for half-maximal activation; and *s* is the slope factor. As shown in Fig. 4c, the *V*_1/2_ in the case of the 3 mM K^+^ solution was − 117.7 ± 3.4 mV (*n  *= 6). This value was not significantly different from that in the case of the 5.4 mM K^+^ solution (− 118.5 ± 2.9 mV, *n *= 6). These results indicate that approximately 18% of the *I*_HCN_ was activated at the RMP of HCN2-Tg mouse myocytes in the 3 mM [K^+^]_o_ solution.

### Ventricular ectopy induced by hypokalemia in HCN2-Tg mice

**Fig. 5:**
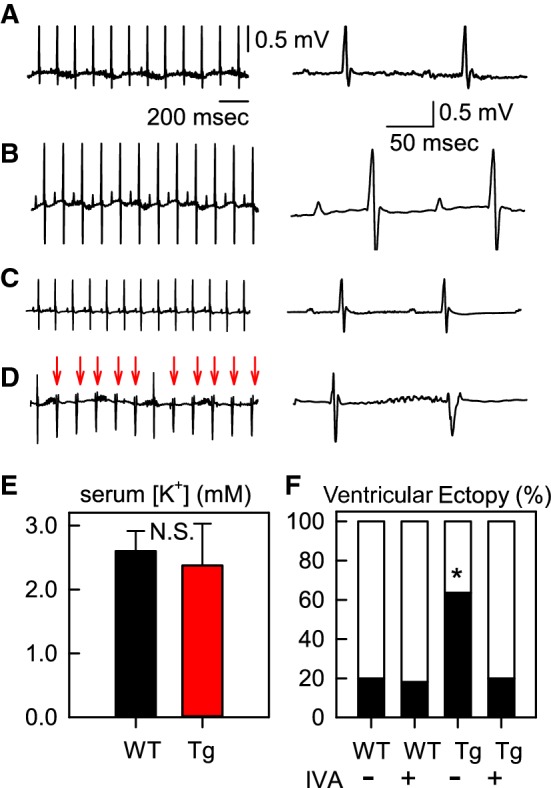
The electrocardiogram (ECG) recordings of WT and HCN2-Tg mice after they were fed a K^+^-free diet for 6 weeks. **a** ECG of WT mouse under control conditions. **b** ECG of a WT mouse after 6 weeks on a K^+^-free diet. **c** ECG of HCN2-Tg mouse under control conditions. **d** ECG of a HCN2-Tg mouse after 6 weeks on a K^+^-free diet. Ventricular ectopy (VE) is indicated by red arrows in all cases. In **a**–**d**, the panels to the left show the expanded traces. **e** Serum K^+^ concentrations measured after the ECG recordings.* N.S.* Not signficant. **f** Summary of the incidence of VE. In each experiment, ECGs were recorded for 5 min to confirm the incidence of VE. Asterisk indicates significant difference (*χ*^2^ test, *p* < 0.01) (color figure online)

We next examined whether hypokalemia could induce ventricular arrhythmia in HCN2-Tg mice in vivo. As we reported previously, and as seen in Fig. 5a, b, ECG waveforms of WT and HCN2-Tg mice did not differ significantly under control conditions [9]. To record ECGs under hypokalemic conditions, we raised the WT and HCN2-Tg mice on a K^+^-free diet for 6 weeks [12], following which we recorded the ECGs and then measured serum K^+^ concentrations. The magnitudes of hypokalemia in the WT and HCN2-Tg mice again did not differ significantly (Fig. 5c: WT, black bar, 2.6 ± 0.3 mM; HCN2-Tg, red bar, 2.4 ± 0.6 mM; *n* = 3). As summarized in Table 3, the ECG parameters (RR, PQ, QT, and QTc) were significantly prolonged by hypokalemia in both WT and HCN2-Tg mice, although no significant difference was found between the ECG parameters of WT and HCN2-Tg mice irrespective of the hypokalemia.

**Table 3:** Electrocardiogram parameters of wild-type mice and transgenic mice overexpressing the HCN2 channel

ECG parameters	Test conditions^a^	WT mice	HCN2-Tg mice
RR (s)	CTR	0.121 ± 0.013	0.113 ± 0.06
	Hypokalemia	0.174 ± 0.015*	0.139 ± 0.022*
PQ (s)	CTR	0.039 ± 0.008	0.038 ± 0.005
	Hypokalemia	0.048 ± 0.004*	0.047 ± 0.005*
QT (s)	CTR	0.042 ± 0.006	0.042 ± 0.006
	Hypokalemia	0.072 ± 0.005*	0.067 ± 0.003*
QTc (s)^b^	CTR	0.119 ± 0.017	0.125 ± 0.007
	Hypokalemia	0.189 ± 0.022	0.179 ± 0.018*

ECG, Electrocardiogram

*Significant difference between the values of CTR and hypokalemia at* p* < 0.05 (*t* test) (*n* = 3–6)

^a^The control ECGs (CTR) were recorded before the mice were started on the K^+^-free diet. The ECGs in the hypokalemic condition (Hypokalemia) were recorded after the mice had been fed a K^+^-free diet for 6 weeks

^b^QTc was calculated according to Bazett’s formula

Notably, ventricular ectopy (VE) was recorded in 63.6% of HCN2-Tg mice, as indicated by the red arrows in Fig. 5d (*n * = 7 of 11 mice). Similar VE was also observed in 20.0% of the WT mice (data not shown, 2 of 10 mice). We have previously reported that the VE induced by β-adrenergic stimulation in HCN2-Tg mice was successfully inhibited by IVA [7]. Likewise, in the present study the oral application of IVA reduced the incidence of VE in HCN2-Tg mice (to 20.0%, *n  *= 2 of 10). As summarized in Fig. 5f, the incidence of VE induced by the K^+^-free diet was significantly higher in the HCN2-Tg mice not subjected to IVA treatment (*χ*^2^ test, *p  *< 0.01). Lethal arrhythmias, such as ventricular tachycardia (VT) or ventricular fibrillation (VF) were observed in neither the WT *(n * = 0 of 11) nor the HCN2-Tg (*n  *= 0 of 10) mice.

## Discussion

Hypokalemia is known to have a pro-arrhythmic effect even in the normal heart. In heart failure patients, activation of the renin–angiotensin–aldosterone system is known to induce hypokalemia. Diuretic treatment also exacerbates hypokalemia [4]. However, the effects of hypokalemia on failing hearts that undergo electrophysiological remodeling remain poorly understood. We had previously reported that HCN channels were re-expressed in the ventricular myocytes of a mouse model of heart failure [6]. In the present study, we demonstrated that HCN2-overexpressing ventricular myocytes showed a higher vulnerability to arrhythmia even under mild, commonly observed hypokalemic conditions.

Serum K^+^ concentration is strictly regulated within a narrow range (3.5–5.0 mM) by many organs in the human body, and a serum K^+^ concentration of < 3.5 mM is clinically defined as hypokalemia [4]. Hypokalemia promotes ventricular arrhythmias, such as VE, Torsades de Pointes, VT, and VF, due to its effect of increasing the electrical instability of cardiac myocytes, mainly by two mechanisms. First, hypokalemia prolongs the APD and induces early after-depolarization (EAD) by decreasing the repolarization reserve of the ventricular AP [8, 13]. Second, hypokalemia inhibits the activity of Na^+^–K^+^ ATPase (NKA) and decreases the amplitude of outward pump currents, which also contribute to the repolarization reserve [14]. It has also been reported that the inhibition of NKA promotes intracellular Na^+^ accumulation and intracellular Ca^2+^ overload via the consequent decrease in the driving force of the Na^+^–Ca^2+^ exchanger (NCX) [2, 14, 15]. Under this condition, spontaneous Ca^2+^ sparks are increased by calmodulin kinase II (CaMKII) via the phosphorylation of the ryanodine receptor (RyR), inducing the inward current carried by NCX [14, 15]. CaMKII may also activate the late Na^+^ current (*I*_Na_) and l-type Ca^2+^ current (*I*_Ca–L_) [14]. EAD has been reported to be induced by these mechanisms 10–50 min after the ex vivo perfusion of rabbit hearts with a hypokalemic solution [14]. The Ca^2+^ overload may also promote ventricular arrhythmia by activating the transient receptor potential channels [16].

In addition to the above mechanisms, based on our results we propose a novel mechanism underlying hypokalemia-induced arrhythmia in failing hearts, whereby the HCN channels overexpressed in the heart are constitutively activated at hyperpolarized RMPs under hypokalemic conditions, resulting in destabilization of the RMP. As shown in Fig. 2, SAP was induced in 55% of HCN2-Tg mouse myocytes; in the remaining HCN2-Tg mouse myocytes, the RMP in the hypokalemic solution was further hyperpolarized by IVA treatment. It should be noted that Chen et al. reported that when HCN2 and Kir2.1 were co-expressed in HEK293 cells, the RMP of these cells oscillated between − 64 and − 34 mV, depending on the balance between the expression levels of HCN2 and Kir2.1 [17]. Based on the same rationale, we suggest that the diversity of hypokalemic responses in HCN2-Tg mouse myocytes may be due to variation in the HCN2 expression levels, such that when the oscillation of RMP exceeds the threshold of *I*_Na_, HCN2-Tg mouse myocytes may start to fire. The SAPs that follow may be due to the activation of *I*_Ca–L_; a similar mechanism for EADs has been reported in rabbit myocytes [8]. The reversal potential of *I*_HCN_ was approximately − 35 mV in the hypokalemic solution. Therefore, the inward *I*_HCN_ should decrease the repolarization reserve at membrane potentials that are more negative than − 35 mV. In fact, we observed that the APD of quiescent HCN-Tg mouse myocytes was significantly longer under hypokalemic conditions. This finding agrees well with that of our previous study in which *I*_HCN_ activated by β-adrenergic stimulation was shown to have a similar effect on the APD of HCN2-Tg mouse myocytes [9].

### Limitations of the present study

In this study, we employed the ruptured whole-cell patch method using a pipette solution containing EGTA in order to focus on the arrhythmogenic role of the ion channels expressed on the plasma membrane. However, it has been reported that the plateau phase of the AP of murine ventricular myocytes is at approximately − 40 mV, and is generated by the NCX current [18]. Therefore, the participation of the intracellular Ca^2+^ transient in the configuration of the AP may have been underestimated; i.e., when the intracellular Ca^2+^ transient was kept intact, APD may have been more prominently prolonged under hypokalemic conditions. At the given composition of the bathing solution, the inward *I*_HCN_ current was mainly carried by external Na^+^. Therefore, under hypokalemic conditions, it appears likely that the constitutive activation of *I*_HCN_ may lead to intracellular Na^+^ accumulation and, consequently, Ca^2+^ overload in HCN2-Tg mouse myocytes. Future studies should measure the intracellular Na^+^ and Ca^2+^ transients of HCN2-Tg mouse myocytes under hypokalemic conditions.

The APD of murine ventricular myocytes is much shorter than that of myocytes of larger animals, mainly due to the different expression pattern of repolarizing K^+^ channels. The repolarization reserve of rabbit myocytes reportedly consists of *I*_Kr_ (Kv11.1) and *I*_K1_; the conductance of both these components is highly sensitive to [K^+^]_o_ [19]. In contrast, *I*_Kr_ is not expressed in murine myocytes. The voltage-gated K^+^ channels expressed in murine myocytes are: Kv4.2 (fast component of transient outward current; *I*_to, fast_), Kv1.4 (slow component of transient outward current; *I*_to, slow_), Kv1.5 (slow delayed rectifier K^+^ current-1; *I*_K, slow1_), and Kv2.1 (slow delayed rectifier K^+^ current-2; *I*_K, slow2_) [19]. The conductance of Kv1.4 [20], Kv1.5 [21], and Kv2.1 [22] are reportedly less sensitive to [K^+^]_o_ than that of *I*_K1_ [23] or *I*_Kr_ [24]. Therefore, under hypokalemic conditions, the decrease in the repolarization reserve in murine hearts may not be as remarkable as that in the hearts of larger animals. For this reason, lethal arrhythmia may not have been induced in vivo by hypokalemia. Future studies should examine the effects of hypokalemia in the myocytes isolated from hypertrophied hearts of larger animals, such as rabbits, following electrophysiological remodeling.
